# O.2.2-5 Health promotion in rural areas. Findings from two German projects on physical activity promotion among disadvantaged populations

**DOI:** 10.1093/eurpub/ckad133.120

**Published:** 2023-09-11

**Authors:** Tobias Fleuren, Maike Till, Heiko Ziemainz, Karim Abu-Omar

**Affiliations:** Friedrich-Alexander-Universität Erlangen-Nürnberg, Germany; tobias.fleuren@fau.de; Friedrich-Alexander-Universität Erlangen-Nürnberg, Germany; tobias.fleuren@fau.de; Friedrich-Alexander-Universität Erlangen-Nürnberg, Germany; tobias.fleuren@fau.de; Friedrich-Alexander-Universität Erlangen-Nürnberg, Germany; tobias.fleuren@fau.de

## Abstract

**Purpose:**

Communities play a crucial role for health promotion, especially for disadvantaged groups. At the same time, rural communities face specific challenges when planning and conducting health promotion projects. This presentation focuses on the challenges and opportunities rural communities experienced in two German projects for physical activity promotion.

**Methods:**

Data were obtained from city administration staff participating in the GESTALT and the BIG projects (N = 13). Data were gathered via three storywall surveys. In addition, a real-world lab with city-administration staff, representatives of sickness funds and researchers was used to follow the communities over time and consult them on project implementation.

**Results:**

The challenges and opportunities occurred can be categorized in five domains: Infrastructure/mobility: Rural areas are often not well connected to public transport. This can hinder people to attend exercise classes. Voluntarily organized transport services or neighborhood assistance might help overcoming this challenge.

**Networking:**

Networking is a double-edged sword. Some staff reported to experience benefits of already existing contacts to relevant stakeholder due to closely-knit communities. Others reported that it is challenging to get in touch with the network partners in the rural areas.

**Durability:**

In some cases, the project funding was seen as a window of opportunity and a starting point for developing new structures for health promotion.

**Range of offers:**

A need of exercise classes in rural areas resulted in the success of the projects. However, the implementation of new programs and activities can be time-consuming. Especially in rural districts, solutions often are planned and implemented on a very small scale.

**Implementation:**

Regarding implementation, there are several challenges to overcome. The communities lacked gyms and qualified instructors for the classes. Reaching the target population was another challenge.

**Conclusions:**

Rural communities face unique as well as general challenges when implementing exercise programs, which can impact success of project implementation. However, rural structures can also result in opportunities for the participating communities.

**Support/Funding Source:**

Funded by the German Federal Centre for Health Education (BZgA) on behalf of and with funds from the statutory health insurance companies according to § 20a SGB V.

